# VGLUT2 Trafficking Is Differentially Regulated by Adaptor Proteins AP-1 and AP-3

**DOI:** 10.3389/fncel.2017.00324

**Published:** 2017-10-26

**Authors:** Haiyan Li, Magda S. Santos, Chihyung K. Park, Yuriy Dobry, Susan M. Voglmaier

**Affiliations:** Department of Psychiatry, School of Medicine, Weill Institute for Neurosciences, Kavli Institute for Fundamental Neuroscience, University of California, San Francisco, San Francisco, CA, United States

**Keywords:** glutamate, vesicular glutamate transporter, VGLUT, synaptic vesicle, endocytosis, exocytosis

## Abstract

Release of the major excitatory neurotransmitter glutamate by synaptic vesicle exocytosis depends on glutamate loading into synaptic vesicles by vesicular glutamate transporters (VGLUTs). The two principal isoforms, VGLUT1 and 2, exhibit a complementary pattern of expression in adult brain that broadly distinguishes cortical (VGLUT1) and subcortical (VGLUT2) systems, and correlates with distinct physiological properties in synapses expressing these isoforms. Differential trafficking of VGLUT1 and 2 has been suggested to underlie their functional diversity. Increasing evidence suggests individual synaptic vesicle proteins use specific sorting signals to engage specialized biochemical mechanisms to regulate their recycling. We observed that VGLUT2 recycles differently in response to high frequency stimulation than VGLUT1. Here we further explore the trafficking of VGLUT2 using a pHluorin-based reporter, VGLUT2-pH. VGLUT2-pH exhibits slower rates of both exocytosis and endocytosis than VGLUT1-pH. VGLUT2-pH recycling is slower than VGLUT1-pH in both hippocampal neurons, which endogenously express mostly VGLUT1, and thalamic neurons, which endogenously express mostly VGLUT2, indicating that protein identity, not synaptic vesicle membrane or neuronal cell type, controls sorting. We characterize sorting signals in the C-terminal dileucine-like motif, which plays a crucial role in VGLUT2 trafficking. Disruption of this motif abolishes synaptic targeting of VGLUT2 and essentially eliminates endocytosis of the transporter. Mutational and biochemical analysis demonstrates that clathrin adaptor proteins (APs) interact with VGLUT2 at the dileucine-like motif. VGLUT2 interacts with AP-2, a well-studied adaptor protein for clathrin mediated endocytosis. In addition, VGLUT2 also interacts with the alternate adaptors, AP-1 and AP-3. VGLUT2 relies on distinct recycling mechanisms from VGLUT1. Abrogation of these differences by pharmacological and molecular inhibition reveals that these mechanisms are dependent on the adaptor proteins AP-1 and AP-3. Further, shRNA-mediated knockdown reveals differential roles for AP-1 and AP-3 in VGLUT2 recycling.

## Introduction

Vesicular glutamate transporters (VGLUTs) in the synaptic vesicle (SV) membrane load glutamate into SVs for exocytotic release, the primary mechanism for information transfer in the nervous system (Bellocchio et al., 2000; Takamori et al., 2000, 2001). The two main isoforms, VGLUT1 and 2, account for the exocytotic release of glutamate by most well-established excitatory neurons (Fremeau et al., 2001; Takamori et al., 2001; Varoqui et al., 2002). VGLUT1 and 2 exhibit different spatiotemporal patterns of expression. VGLUT2 is highly expressed early in development, while VGLUT1 expression increases during brain maturation (Boulland et al., 2004; Nakamura et al., 2005). In the adult, VGLUT1 and 2 exhibit a complementary pattern of expression, with VGLUT1 predominating in the neocortex, hippocampus, and cerebellar cortex, while VGLUT2 predominates in subcortical brainstem nuclei, thalamic nuclei, and cerebellar deep nuclei. Thus VGLUT isoforms define discrete anatomical glutamatergic pathways (Fremeau et al., 2001; Herzog et al., 2001; Takamori et al., 2001; Varoqui et al., 2002). VGLUT1 is expressed in cortico-cortical glutamatergic systems while VGLUT2 is expressed in thalamocortical systems. In general, these glutamatergic systems are associated with differences in the source of information, with VGLUT2 pathways carrying sensory information from the external world transmitted through the thalamus, while VGLUT1 carries mnemonic information from other areas of cortex. The precise balance of these glutamatergic pathways is necessary to integrate information and coordinate output behavior (Carlsson et al., 1997; Jones, 2002; Moutsimilli et al., 2008).

At the physiological level, VGLUT expression in the adult brain generally correlates with the probability of transmitter release and the potential for plasticity. The VGLUT1 isoform is expressed in brain regions that exhibit a higher potential for plasticity (cortex and hippocampus), whereas VGLUT2 is expressed in regions that transmit signals with higher fidelity (subcortex) (Fremeau et al., 2001, 2004b; Varoqui et al., 2002). Expression of a VGLUT1 or VGLUT2 isoform in transfected neurons can produce distinct physiological characteristics, defining the properties of the synapse expressing the protein. Synapses expressing VGLUT1 exhibit a lower initial probability of release and show synaptic facilitation, while synapses expressing VGLUT2 exhibit a higher probability of release and synaptic depression (Fremeau et al., 2004a; Weston et al., 2011).

The basic properties of glutamate transport mediated by the two isoforms are essentially identical, first suggesting that the biochemical transport function of the proteins was unlikely to underlie the physiological differences of synapses expressing VGLUT1 or VGLUT2. Subtle differences in the steady-state localization observed in transfected PC12 cells, with VGLUT1 exhibiting a more peripheral and VGLUT2 a diffuse distribution, initially suggested differences in protein trafficking (Fremeau et al., 2001). Although previous studies assumed all SV proteins recycle equivalently, we observed that VGLUT1 and 2 exhibit kinetic differences in their trafficking, indicating that in addition to their role in loading glutamate into SVs, the proteins themselves control their recycling (Foss et al., 2013). Despite a high degree of conservation in the rest of the protein, VGLUT1 and 2 diverge in their N- and C-termini. The C-terminus of VGLUT1 contains two polyproline (PP) domains not present in VGLUT2. The interaction of a PP domain with the endocytic protein endophilin contributes to fast recycling (Voglmaier et al., 2006) and synaptic facilitation of VGLUT1 (Weston et al., 2011). The effect of endophilin in directing VGLUT1 toward a fast endocytic pathway is dependent on an upstream C-terminal dileucine-like motif (Voglmaier et al., 2006).

Dileucine motifs consist of two hydrophobic residues preceded by acidic residues four and/or five positions upstream, and are essential for driving clathrin-mediated endocytosis of cargo proteins by recruiting adaptor proteins (APs) (Takei et al., 1996; Robinson and Bonifacino, 2001). While VGLUT1 and VGLUT2 share a similar C-terminal dileucine-like motif, VGLUT1 trafficking is directed by multiple regulatory elements in both the N- and C-termini (Foss et al., 2013). In contrast, VGLUT2 trafficking appears to completely rely on the sorting signal in the C-terminus. However, little is known about the mechanisms underlying VGLUT2 recycling, thus we now examine isoform-specific trafficking of VGLUT2-pHluorin in real time.

## Materials and Methods

### Reagents

Bafilomycin 1A was obtained from Calbiochem (San Diego, CA, United States). CPP [3-(2-carboxypiperazin-4-yl) propyl-1-phosphonic acid] and CNQX (6-cyano-7-nitroquinoxaline-2,3-dione) were purchased from Tocris Bioscience (Ellisville, MO, United States). FM4-64 was obtained from Biotium (Hayward, CA, United States). Brefeldin A (BFA) was purchased from LC Laboratories. Antibody against synaptophysin was obtained from Invitrogen/Life Technologies (Grand Island, NY, United States). Mouse anti-SV2 antibody was a gift of R. Kelly (UCSF) and rabbit anti-VGLUT1 antibody was a gift of R. Edwards (UCSF). Antibodies against AP-1γ, AP-2α, and β-NAP were purchased from BD Bioscience. Secondary antibodies conjugated to FITC, Cy3, or Cy5 were from Jackson ImmunoResearch (West Grove, PA, United States). Secondary antibodies conjugated to HRP were from GE Healthcare Life Sciences. All other chemicals were from Sigma–Aldrich (St. Louis, MO, United States). Cell culture reagents were from Life Technologies, unless otherwise noted.

### Molecular Biology

VGLUT2-pH was constructed similarly to VGLUT1-pH with ecliptic pHluorin inserted in the first luminal loop between Gly-107 and Gly-108 of rat VGLUT2 flanked by the same linker sequences used in VGLUT1-pH (Voglmaier et al., 2006). Standard PCR-directed overlap extension mutagenesis was used to modify VGLUT2-pH to make the FI/AA and FI/GG mutations in the pCAGGS vector. To make chimeric VGLUT1-pH with a VGLUT2 C-terminal tail (VGLUT1/2-pH) and VGLUT2-pH with a VGLUT1 C-terminal tail (VGLUT2/1-pH), the C-termini were swapped after the last transmembrane domain. An NheI site was introduced by PCR mutagenesis without changing the amino acid sequence GCCTCG (Ala Ser)->GCC***AGC*** (Ala Ser) within the conserved F_489_ASGE_493_ in rat VGLUT1, and F_497_ASGE_501_ in rat VGLUT2, and standard subcloning techniques were used to swap the tail fragments. All cDNAs were subcloned into pCAGGS for neuronal expression under the control of a modified chicken actin promoter. For GST-fusion recombinant protein expression, VGLUT2 C-terminal constructs were generated by PCR and subcloned into pGEX vectors (GE Healthcare Life Sciences).

### RNA Interference Knockdown

As described previously (Foss et al., 2013; Santos et al., 2013), lentiviral constructs expressing shRNA to rat AP-1γ (AP-1A shRNA, 5′-ACCGAATTAAGAAAGTAGT-3′) (Dugast et al., 2005; Cheung and Cousin, 2012), or rat AP-3δ1 (5′-CATGGATCATGACCAAGAA-3′) (Asensio et al., 2010) were made in a pFHUBW vector (gift from R. Edwards, UCSF), a variant of pFHUGW containing the monomeric blue fluorescent protein mTagBFP in place of GFP (Lois et al., 2002). The shRNA-resistant AP-1γ rescue construct was made by introducing a silent mutation to the AP-1γ target sequence: ACCGAAT***C***AAGAAAGTAGT; and the shRNA-resistant AP3-δ1 plasmid was generated by introducing a silent mutation to the AP-3δ1 target sequence: 5′-CATGGATCA***C***GACCAAGAA-3′. Both shRNA-resistant cDNAs were subcloned into a pCAGGS-IRES2-mCherry vector. Recombinant lentiviruses were produced in HEK293T cells as described previously (Foss et al., 2013; Santos et al., 2013). Each batch of virus is titered, and tested for efficacy and specificity for AP-1, AP-2, or AP-3 by Western analysis of infected hippocampal neurons. All PCR generated products were confirmed by sequencing to ensure faithful amplification.

### GST Pull-Downs

Extracts from rat brains were solubilized in 100 mM Tris-HCl, pH 7.5, 150 mM NaCl, 1 mM EGTA, and 1% Triton X-100 containing protease inhibitors (1 mg/ml E64, 2 mg/ml aprotinin, 2 mg/ml leupeptin, 2 mg/ml pepstatin, and 20 mg/ml PMSF), sedimented at 20,000 × *g* for 45 min at 4°C, and the supernatant (∼400 mg total protein) incubated with 200 μg of GST-fusion proteins immobilized on glutathione-Sepharose at 4°C for 2 h with rotation, as described (Santos et al., 2013). After pelleting, the beads were washed and bound proteins were detected by immunoblot analysis using mouse monoclonal anti-adaptin γ (1:500), anti-adaptin α (1:1000), and β-NAP (1:250) antibodies. ImageJ was used to determine the intensity of bands using the intensity of the respective fusion protein loaded on the same lane (revealed by Ponceau S staining) to normalize the signal.

### Primary Neuronal Culture, Transfection, and Immunofluorescence

Hippocampi from embryonic days 19–20 rats of either sex were dissected and dissociated as previously described (Li et al., 2011). Neurons were transfected using the Basic Neuron SCN Nucleofector kit, according to manufacturer’s directions (Lonza, Walkersville, MD, United States). Neurons transfected by nucleofection express similar, moderate levels of protein (Li et al., 2005). Cells were maintained in Neurobasal media supplemented with 1% heat inactivated fetal bovine serum (FBS), 2% NeuroMix growth supplement (PAA, Dartmouth, MA, United States), 2 mM GlutaMax, 15 mM NaCl, and 10 μg/ml Primocin or MycoZap antibiotic (Lonza) and imaged at 14–21 days *in vitro* (DIV). For thalamic cultures, ¼ of the growth media was replaced with serum-free media at DIV 3, and ½ at DIV 5, 7, and 9 (Kanagasabapathi et al., 2011). 5-fluoro-2′-deoxyuridine (10 μM final concentration) was added at DIV 3–5 as a mitotic inhibitor to control glial growth. For RNAi knockdown (KD) experiments, cells were infected at DIV 7 and imaged at DIV 14–18. All work with animals was approved by and conducted under the supervision and guidance of the Institutional Care and Use Committee of the University of California, San Francisco, Office of Ethics and Compliance. For immunostaining of transfected cultures, cells were fixed in 4% paraformaldehyde for 5 min, then in cold methanol for 5 min at -20°C, permeabilized, and blocked in phosphate buffered saline (PBS) containing 0.02% saponin, 1% fish gelatin, and 5% BSA, and then stained with rabbit anti-VGLUT1, mouse or rabbit anti-synaptophysin, or mouse anti-SV2, followed by appropriate secondary antibodies conjugated to FITC, Cy3, or Cy5. Cells were imaged using confocal laser microscopy (Zeiss LSM510). For immunostaining of endogenous VGLUTs, cultured hippocampal and thalamic neurons were fixed as above, and permeabilized and blocked in PBS containing 0.1% Triton X-100 and 5% FBS. Antibody staining was performed with guinea pig anti-VGLUT1 (1:5000, Chemicon AB5905), rabbit anti-VGLUT2 (1:1000, Synaptic Systems 135103), and mouse anti-synaptophysin (1:2000, Sigma–Aldrich S5768), followed by appropriate secondary antibodies conjugated to FITC, Cy3, or Cy5. Images were acquired on a QuantEM CCD camera (Photometrics) with the appropriate filter for each antibody. Image analysis was performed with MetaMorph (Molecular Devices) using the built in auto threshold for light objects function to correct for differences in background and out of focus fluorescence. Pixels above the threshold were selected at random by centering 4 × 4 pixel boxes over the boutons stained with anti-synaptophysin antibody, constituting the regions of interest (ROIs). The synaptophysin^+^ ROIs were then loaded onto the corresponding images obtained with either the VGLUT1 or VGLUT2 antibody and the average fluorescence for each ROI was automatically determined. Percentage of synaptophysin^+^, VGLUT1 or 2^+^ was calculated by dividing the number of VGLUT1 or 2^+^ puncta by the number of synaptophysin^+^ puncta, multiplied by 100.

### Live Cell Imaging

Live cell imaging was performed essentially as described previously (Voglmaier et al., 2006). Coverslips with transfected hippocampal neurons were mounted in a rapid switching, laminar-flow perfusion and stimulation chamber (Warner Instruments, Holliston, MA, United States) on an inverted epifluorescence microscope (Nikon, Melville, NY, United States) and imaged at room temperature using a 63X oil objective (NA = 1.4). Cells were imaged in modified Tyrode’s solution pH 7.4 (in mM: 119 NaCl, 10 HEPES-NaOH, 30 glucose, 2.5 KCl, 2 CaCl_2_, 2 MgCl_2_) containing 10 μM each of the glutamate receptor inhibitors CNQX and CPP. For readily releasable pool of vesicles (RRP) experiments, Ca^2+^ concentration was increased to 4 mM (and NaCl decreased to 117 mM), or kept at 2 mM. Tyrode’s solution at pH 5.5 was prepared by replacing HEPES with MES. Hypertonic sucrose was prepared by adding 300 mM sucrose to modified Tyrode’s solution. Electrical stimulation to elicit action potentials (Gandhi and Stevens, 2003) was applied using an A310 Accupulser (WPI, Sarasota, FL, United States) at 5–100 Hz with 1 ms bipolar current pulses through platinum-iridium electrodes, to yield fields of 5–10 V/cm across the chamber. Cells were illuminated using a Xenon lamp (Sutter Instruments, Novato, CA, United States) with either a 470/40-nm excitation and a 525/50-nm emission filter (for GFP), a 470/40-nm excitation and 630/75 nm emission filter (for FM4-64) (Chroma, Bellows Falls, VT, United States). Images were acquired on a QuantEM CCD camera (Photometrics, Tucson, AZ, United States), exposing each fluorophore for 300 ms for images collected every 1 s, 3 s or 6 s. MetaMorph software was used to control data collection and to perform offline analysis (Universal Imaging, Sunnyvale, CA, United States). The total pool size was determined using Tyrode’s solution with 50 mM NH_4_Cl (NaCl reduced by 50 mM). To measure exocytosis alone, cultures were incubated in modified Tyrode’s solution containing 0.5–1 μM bafilomycin A for 30 s before imaging in the same medium. To assess exocytosis with FM4-64, cultures were incubated in modified Tyrode’s solution containing 15 μM FM4-64 and stimulated at 10 Hz for 60 s, followed by continued incubation in the same medium for an additional 60 s. After extensive washing, for 10–15 min at a rate of 6 ml/min in modified Tyrode’s solution without FM4-64, the FM dye was unloaded by stimulation at 10 Hz for 90 s. Transfected boutons were identified by visualization of VGLUT2-pH puncta in the presence of 50 mM NH_4_Cl, with washout before FM4-64 loading. For BFA treatment to block AP-1 and AP-3 pathways, cultures were pretreated in complete Neurobasal medium containing 10 μg/ml BFA for 30 min at 37°C before imaging in modified Tyrode’s solution containing the same concentration of BFA.

### Data Analysis

As described previously (Voglmaier et al., 2006; Li et al., 2011), MetaMorph software was used to quantify the average fluorescence of ROI at synaptic sites at manually selected 4 × 4 pixel boxes placed over the center of boutons. Criteria to select ROIs use manual image segmentation, including thresholding by fluorescence above background, morphology, size of ∼1–2 um^2^, partially quenched fluorescence which increases upon application of NH_4_Cl or electrical stimulation, one optical center of mass in 50 mM NH_4_Cl with separation from other nearby sources of light scatter, and a stable pre-stimulus baseline fluorescence and post-stimulus plateau or recovery. All regions fitting these criteria on the analyzed region of the coverslip are analyzed. Each neuronal culture is tested with a control construct [wild-type (WT) VGLUT1-pH] for activity-dependent fluorescence response and recovery. The average fluorescence of three 4 × 4 pixel ROIs without cellular elements was subtracted as background. Baseline values from the first five frames (before stimulation) were averaged as initial fluorescence F_0_, and the dynamics of fluorescence intensity expressed as fractional change (ΔF) over initial fluorescence. For normalized measurements, the average pHluorin fluorescence over individual boutons was normalized to either the peak fluorescence in each trace, or the total fluorescence as visualized by application of modified Tyrode’s solution containing 50 mM NH_4_Cl to alkalinize all synaptic compartments. To calculate the fraction of transporter on the cell surface, we take advantage of the fact that only in protein stranded at the cell surface does pHluorin face the outside of the cell. We first quench surface fluorescence with Tyrode’s solution at pH 5.5, with MES replacing HEPES, added to the outside of the cell, to measure background fluorescence (average fluorescence intensity of five frames). Background fluorescence is subtracted from the fluorescence intensity in standard pH 7.4 Tyrode’s solution (before stimulation), reflecting surface fluorescence intensity. The difference is divided by total fluorescence determined in the presence of 50 mM NH4Cl (total protein) (Foss et al., 2013). Fluorescence measurements from 16 to 200 boutons per coverslip were averaged and the means from 4 to 22 coverslips from at least two independent cultures were averaged. Data are presented as means ± SEM. Significance of differences between groups was assessed by two-tailed, unpaired *t*-test at *p* < 0.05 where appropriate (GraphPad Prism).

To measure the rate of exocytosis and to determine the total amount of transporter that underwent exocytosis, cells were imaged in modified Tyrode’s solution containing bafilomycin. The fraction of transporter that undergoes exocytosis in response to 10 Hz 90 s stimulation (recycling pool, RP) was measured as the fraction of the total pool (Foss et al., 2013). The rate of exocytosis [(ΔF/F_0_)/s] was estimated from a linear fit to the increase in pHluorin fluorescence during the initial 15 s of stimulation in the presence of bafilomycin. To calculate the proportion of both VGLUT1-pH and VGLUT2-pH in the RRP by 20 action potential electrical stimulation, neurons were stimulated in the presence of bafilomycin and the first five frames after stimulation were averaged and normalized to the total fluorescence. To calculate the proportion of VGLUT1-pH and VGLUT2-pH in the RRP by challenge with hypertonic sucrose, neurons were stimulated with Tyrode’s solution containing 300 mM sucrose for 6 s in the presence of 1 μM of bafilomycin to prevent reacidification of the internalized vesicles, and imaged in the absence of sucrose (to avoid distortion by changes in refractive index) both before and after stimulation (Nemani et al., 2010). RRP size was also evaluated using an alternate stimulus of 90 action potentials at 30 Hz (Pyle et al., 2000). To determine the percentage of decline from peak during stimulation [Δ(ΔF/F_0_)], the fluorescence recorded at the last time point of stimulation (60 s) was subtracted from peak fluorescence, and the difference expressed as a percentage of peak fluorescence. For measurements of endocytosis after stimulation, the time course of fluorescence decay at each bouton after the initial 3 s was fit with a single exponential (GraphPad Prism) (Balaji et al., 2008).

## Results

### VGLUT2 Undergoes Exocytosis and Endocytosis at Different Rates than VGLUT1

**FIGURE 1:**
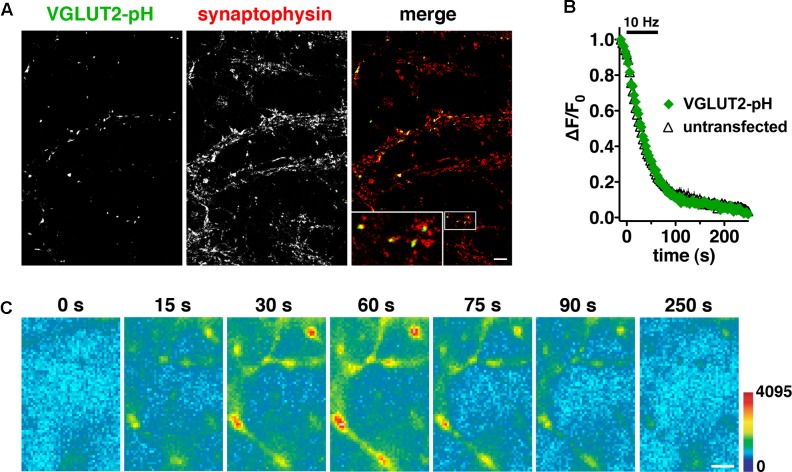
Characterization of a pHluorin-based reporter for studying vesicular glutamate transporter 2 (VGLUT2) recycling in real time. **(A)** VGLUT2-pH fluorescence co-localizes with synaptophysin at synaptic boutons. Hippocampal neurons transfected with VGLUT2-pH were stained with antibody to endogenous synaptophysin, followed by Cy5-conjugated secondary antibody. Inset shows a 9× magnification of the designated box. Scale bar, 10 μm. **(B)** The rate of FM4-64 destaining is not significantly different between boutons from untransfected (black) and transfected (green) neurons. Data are means ± SEM of the change in fluorescence (ΔF), acquired every 3 s, normalized to initial fluorescence (average of the first five data points prior to stimulation, F_0_) over 26–49 boutons per coverslip from four coverslips (n) from two independent cultures for each condition. **(C)** Time-lapse images show the fluorescence changes of VGLUT2-pH in response to neural activity. After onset of a 10 Hz 60 s stimulus, exocytosis of VGLUT2-pH results in a rapid increase in fluorescence (15, 30, and 60 s), followed by fluorescence decay after the termination of stimulation (75, 90, and 250 s) as vesicles undergo endocytosis and reacidification. Color scale is shown to the right. Scale bar, 2 μm.

Differences in recycling may contribute to different physiological properties observed in VGLUT1 and VGLUT2 expressing neurons (Fremeau et al., 2004a; Weston et al., 2011). Indeed we previously observed differences in the time course of fluorescence changes in VGLUT1-pH and VGLUT2-pH (Foss et al., 2013). Here we further explore the trafficking differences and pursue the mechanistic causes. To investigate synaptic trafficking of VGLUT2 in live neurons, we generated the optical probe VGLUT2-pHluorin (VGLUT2-pH) by inserting super-ecliptic pHluorin into the first luminal loop of rat VGLUT2 (Voglmaier et al., 2006; Foss et al., 2013). When expressed in hippocampal neurons, VGLUT2-pH exhibits a punctate distribution with accumulation at synaptic boutons, where it co-localizes with the synaptic markers synaptophysin (**Figure 1A**), SV2, and endogenous VGLUT1 (data not shown). Activity-dependent unloading of the styryl dye FM4-64 from SVs is not affected by expression of VGLUT2-pH, indicating that the tagged transporter does not perturb general features of the vesicle cycle (**Figure 1B**). Like VGLUT1-pH, the fluorescence of VGLUT2-pH is quenched at the acidic pH of SVs (**Figure 1C**, 0 s). Exocytosis induced by electrical stimulation exposes VGLUT2-pH to a neutral extracellular pH, which is visualized as a rapid increase in fluorescence measured at individual boutons (**Figure 1C**, 15–60 s). This fluorescence increase is calcium-dependent (data not shown). Subsequent endocytosis is reflected by a decrease in fluorescence of the reporter, as internalized vesicles are rapidly reacidified (**Figure 1C**, 75–250 s) (Miesenböck et al., 1998; Sankaranarayanan and Ryan, 2000).

**FIGURE 2:**
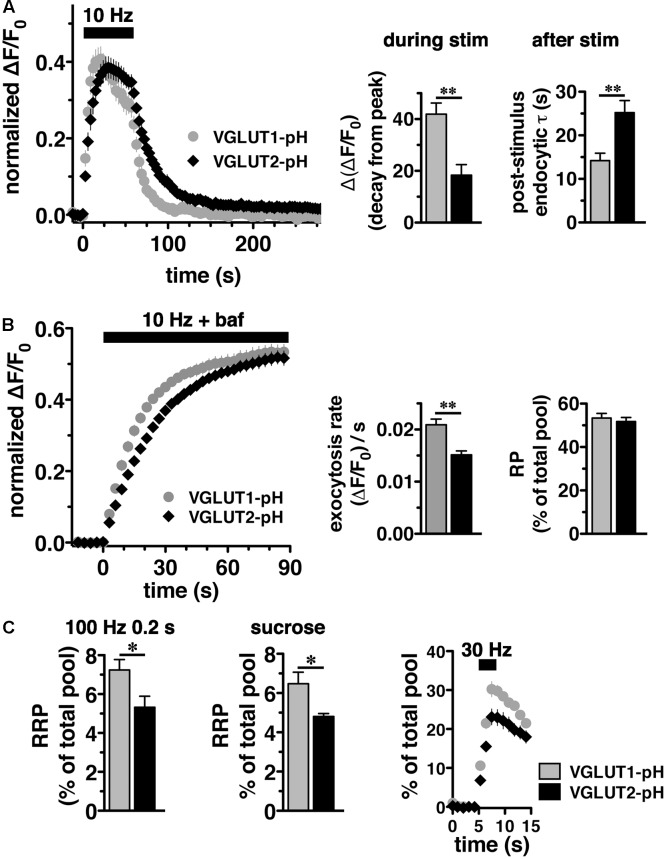
Vesicular glutamate transporter 2-pH recycling differs from VGLUT1-pH. **(A)** *Left panel:* The time course of exo- and endocytosis in response to electrical stimulation at 10 Hz 60 s (bar) is monitored by the increase and decrease in the fluorescence of VGLUT2-pH (black) or VGLUT1-pH (gray) at hippocampal synaptic boutons, acquired every 3 s. Each trace was normalized to the size of the total pool of VGLUT-pH as determined by application of 50 mM NH_4_Cl. The fluorescence of both VGLUT1-pH and VGLUT2-pH increases rapidly upon stimulation and decays with an exponential time course after cessation of the stimulus, consistent with exocytosis followed by endocytosis (left panel). *Middle panel:* The extent of fluorescence decay from peak [Δ(ΔF/F_0_)] during stimulation is less for VGLUT2-pH (black, 18.26 ± 4.13% from peak, *n* = 9) than VGLUT1-pH (gray, 41.90 ± 4.31% from peak, *n* = 11, ^∗∗^*p* < 0.01). *Right panel:* The post-stimulus endocytic rate of VGLUT2-pH (black, τ_decay_ = 25.20 ± 2.77 s) is also significantly slower than that of VGLUT1-pH (gray, τ = 14.18 ± 1.73 s, ^∗∗^*p* < 0.01). **(B)** *Left panel:* To measure exocytosis, hippocampal neurons expressing VGLUT2-pH or VGLUT1-pH were stimulated at 10 Hz for 90 s (bar) in the presence of 0.5 μM bafilomycin (baf). *Middle panel:* VGLUT2-pH exhibits a slower initial rate of exocytosis than VGLUT1-pH, calculated as the linear rate of fluorescence change, acquired every 3 s, from 0 to 15 s (VGLUT2-pH, 0.0151 ± 0.0008, *n* = 9; VGLUT1-pH, 0.0209 ± 0.0011, *n* = 10, ^∗∗^*p* < 0.01). *Right panel:* Both VGLUT1-pH and VGLUT2-pH fluorescence plateau at a similar level in response to 900 action potentials, indicating that there is no significant difference in the total amount of VGLUT-pH released from the recycling pool (RP, VGLUT1-pH, 53.38 ± 2.11%; VGLUT2-pH, 51.73 ± 1.92% of total pool). **(C)** *Left panel:* The fraction of VGLUT2-pH in the readily releasable pool (RRP) is less than VGLUT1-pH, when exocytosis from the RRP is evoked using a stimulus of 20 action potentials at 100 Hz (VGLUT2-pH, 5.32 ± 0.57%, *n* = 10; VGLUT1-pH, 7.24 ± 0.53%, *n* = 11, ^∗^*p* < 0.05), or *middle panel*: by application of hypertonic (300 mM) sucrose in Tyrode’s solution (VGLUT2-pH, 4.81 ± 0.15%, *n* = 6; VGLUT1-pH, 6.48 ± 0.58%, *n* = 5, ^∗^*p* < 0.05). *Right panel:* Similar results are obtained with an alternate stimulus of 30 Hz for 3 s to release RRP. The release of VGLUT2-pH (black, 6.81 ± 0.640% of total pool, *n* = 8) even from the first second of stimulation (30 action potentials) is significantly smaller than that of VGLUT1-pH (gray, 10.58 ± 1.025%, *n* = 8, ^∗∗^*p* < 0.01). For RRP measurements, fluorescence intensity measurements were acquired every 1 s. Data are means ± SEM of ΔF/F_0_ normalized to total fluorescence over at least 38 boutons per coverslip from 8 to 11 coverslips per construct and at least three independent cultures.

We initially observed that VGLUT2-pH responds differently than VGLUT1-pH to intense (40 Hz) stimulation (Foss et al., 2013). To better understand the isoform-specific trafficking of VGLUTs, we here first examine how the two VGLUTs respond to more moderate stimulation. We expressed VGLUT1- and 2-pH reporters in hippocampal neurons in culture and monitored the recycling of the two isoforms in response to 10 Hz 60 s stimulation. Like VGLUT1-pH, VGLUT2-pH exhibits a rapid increase in fluorescence upon electrical stimulation at 10 Hz for 60 s and a decrease after stimulation. After stimulation ends, VGLUT2-pH fluorescence reflects only endocytosis, and decays with an exponential time course that indicates the endocytosis rate. The rate of post-stimulus endocytosis of VGLUT2-pH (τ = 25.20 ± 2.77 s) is significantly slower than VGLUT1-pH (τ = 14.18 ± 1.73 s, ^∗∗^*p* < 0.01) (**Figure 2A**, right panel). The difference in endocytosis rate is not due to differences in VGLUT1-pH and VGLUT2-pH expression at the plasma membrane since the cell surface expression of both isoforms is similar (Foss et al., 2013). Total protein levels, determined by the amount of fluorescence in the presence of 50 mM NH_4_Cl to alkalinize the vesicles, are also not significantly different for the two isoforms (data not shown). During stimulation, the fluorescence reflects the balance of endocytosis and exocytosis (Sankaranarayanan and Ryan, 2001; Voglmaier et al., 2006). The decay from peak fluorescence during stimulation [Δ(ΔF/F_0_)] is smaller for VGLUT2-pH (18.26 ± 4.13% from peak) than VGLUT1-pH (41.90 ± 4.31% from peak, ^∗∗^*p* < 0.01) (**Figure 2A**, middle panel).

We noted that VGLUT2-pH fluorescence rises more slowly than VGLUT1-pH upon stimulation (**Figure 2A**, left panel). The VGLUT1 and VGLUT2 proteins have been shown to affect vesicle release properties (Fremeau et al., 2004a; Weston et al., 2011), so we next examined the exocytosis of VGLUT2-pH relative to VGLUT1-pH in transfected neurons. To measure the rate of exocytosis, we used alkaline trapping with bafilomycin, an inhibitor of the H^+^-ATPase. Bafilomycin added to the recording medium blocks reacidification of vesicles that have undergone exocytosis and taken up the drug, eliminating fluorescence changes due to the endocytic component of SV recycling, to reveal only exocytosis (Sankaranarayanan and Ryan, 2001). Surprisingly, we found that the initial rate of exocytosis of VGLUT2-pH [(ΔF/F_0_)/s = 0.0151 ± 0.0008] in response to 10 Hz 90 s stimulation is slower than that of VGLUT1 [(ΔF/F_0_)/s = 0.0209 ± 0.0011, ^∗∗^*p* < 0.01] (**Figure 2B**, left and middle panels). An alternative model that considers exocytosis as an exponential process also shows that the tau for exocytosis of VGLUT2-pH (τ = 27.16 ± 2.53 s) is slower than VGLUT1-pH (τ = 17.71 ± 2.00 s), ^∗∗^*p* < 0.01.

One possibility that could underlie a difference in the rate of exocytosis would be a difference in the amount of VGLUT1 and VGLUT2 in the vesicle pools undergoing release. We therefore determined the size of the RP and RRP as a fraction of the total pool of VGLUT1-pH and VGLUT2-pH. The total pool was measured by subtraction of the baseline fluorescence (pre-stimulation) from the total fluorescence as visualized in 50 mM NH_4_Cl (Hua et al., 2011; Foss et al., 2013). We measured the size of the RP by using a standard protocol, stimulating at 10 Hz for 90 s in the presence of bafilomycin (**Figure 2B**) (Fernandez-Alfonso and Ryan, 2008). The proportion of total VGLUT2-pH that undergoes exocytosis with this stimulus (RP, 51.73 ± 0.15% of total pool) is similar to that of VGLUT1-pH (53.38 ± 0.21% of total pool) (**Figure 2B**, right panel), suggesting that the faster exocytosis rate of VGLUT1 relative to VGLUT2 is not due to increased release of the transporter from the RP. The reserve pool, measured by the total pool minus the RP, is also therefore not different between VGLUT1 and 2. However, the measurement of the RP with 900 action potentials does not distinguish the vesicles that undergo exocytosis through the pool that is docked and ready to release (RRP) from the entire RP. We considered that the number or clearance of docking sites could influence the rate of exocytosis (von Gersdorff and Matthews, 1999; Schneggenburger et al., 2002; Neher, 2010). The size of the RRP has been shown to be an important determinant of synaptic function (Rosenmund and Stevens, 1996; Dobrunz and Stevens, 1997). Several methods have been developed for estimating the size of the physiological RRP at the pre-synaptic terminal of hippocampal neurons. Eliciting release of RRP vesicles with 100 Hz stimulation for 0.2 s (Ariel and Ryan, 2010), we found that the fraction of VGLUT2-pH that undergoes exocytosis with this stimulus (5.32 ± 0.57%), is smaller than that of VGLUT1-pH (7.24 ± 0.53%, ^∗^*p* < 0.05) (**Figure 2C**, left panel). The smaller fraction of VGLUT2-pH in the RRP relative to VGLUT1 is also shown by a challenge with hypertonic sucrose, an independent approach to estimate the RRP (Stevens and Tsujimoto, 1995; Rosenmund and Stevens, 1996) (VGLUT1-pH, 6.48 ± 0.58% vs. VGLUT2-pH, 4.81 ± 0.15%, ^∗^*p* < 0.05) (**Figure 2C**, middle panel). Since this difference in RRP in the two VGLUT isoforms was not previously detected (Weston et al., 2011), we also evaluated RRP size using an alternate stimulus of 30 Hz for 3 s (Pyle et al., 2000). The fraction of VGLUT2-pH released by 90 action potentials (black, 23.12 ± 2.025% of total pool, the third time point after stimulation) is significantly smaller than that of VGLUT1-pH (gray, 30.23 ± 1.952%, ^∗^*p* < 0.05) (**Figure 2C**, right panel). However, the fraction released by 30 action potentials may be a better estimate of the RRP, than that released by the full 90 action potential stimulus (Stevens and Williams, 2007). We confirm that the fraction of VGLUT2-pH that undergoes exocytosis elicited by the first 30 action potentials, at the first time point 1 s after stimulus onset (black, 6.810 ± 0.640% of total pool) is also significantly smaller than that of VGLUT1-pH (gray, 10.58 ± 1.025%, ^∗∗^*p* < 0.01).

**FIGURE 3:**
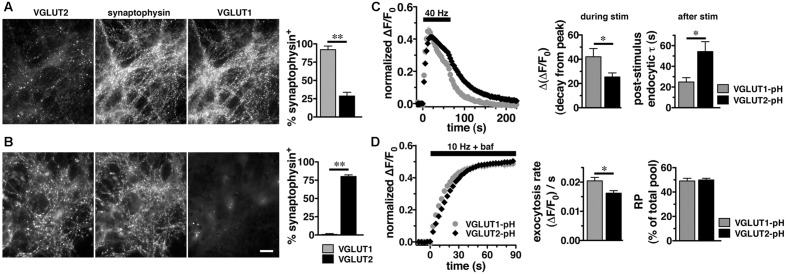
Vesicular glutamate transporter 2-pH retains distinct recycling kinetics from VGLUT1-pH in different neuronal populations. **(A)** Cultured hippocampal neurons were triple stained with antibodies to endogenous VGLUT2, synaptophysin, and VGLUT1. *Right panel:* Quantitation of synaptophysin^+^ puncta that co-localize with antibody to VGLUT1 (92.00 ± 5.05%, *n* = 3) is greater than VGLUT2 (28.67 ± 5.15%, *n* = 4, ^∗∗^*p* < 0.01) in hippocampal cultures. **(B)** In thalamic neurons in culture, 80.00 ± 2.28% of synaptophysin^+^ puncta co-localize with antibody to VGLUT2 (*n* = 5). VGLUT1 staining was virtually undetectable (0.80 ± 0.49%, *n* = 5). Data are the mean ± SEM of 3–5 coverslips from at least two independent cultures. Scale bar, 10 μm. **(C)** *Left panel:* The time course of exo- and endocytosis of VGLUT2-pH (black) or VGLUT1-pH (gray) in response to electrical stimulation in thalamic synaptic boutons is similar to hippocampus. *Middle panel:* The extent of VGLUT2-pH fluorescence decay from peak during stimulation is less for VGLUT2-pH (black, 25.41 ± 3.32%, *n* = 8) than VGLUT1-pH (gray, 42.02 ± 6.85%, *n* = 6, ^∗^*p* < 0.05). *Right panel:* The post-stimulus endocytic rate of VGLUT2-pH (black, τ = 54.32 ± 9.34 s) is also significantly slower than that of VGLUT1-pH (gray, τ = 24.89 ± 4.18 s, ^∗^*p* < 0.05). **(D)** *Left panel:* Exocytosis upon stimulation at 10 Hz for 90 s (bar) is measured in the presence of bafilomycin. *Middle panel:* As in hippocampus, VGLUT2-pH expressed in thalamic neurons exhibits a slower initial rate of exocytosis than VGLUT1-pH, calculated as the linear rate of fluorescence change from 0 to 15 s [(ΔF/F_0_)/s VGLUT2-pH, 0.0162 ± 0.0008, *n* = 8; VGLUT1-pH, 0.0205 ± 0.0012, ^∗^*p* < 0.05, *n* = 9]. *Right panel:* In response to 900 action potentials, fluorescence of VGLUT2-pH and VGLUT1-pH plateaus at a similar level, indicating that there is no significant difference in the total amount of VGLUT-pH released from the RP (VGLUT2-pH, 49.93 ± 1.34% of total pool, VGLUT1-pH, 49.10 ± 2.16%). Data are means ± SEM of ΔF/F_0_, acquired every 3 s, normalized to total fluorescence over at least 24 boutons per coverslip from 6 to 9 coverslips per construct and two independent cultures.

In adult brain, VGLUT2 is expressed predominantly in subcortical neuronal populations, such as thalamus and brainstem, while VGLUT1 predominates in hippocampus and cortex (Hisano et al., 2000; Fremeau et al., 2001; Herzog et al., 2001; Varoqui et al., 2002). While there are some specific differences, endophilins and APs are widely expressed in both hippocampus and thalamus (So et al., 2000; Blumstein et al., 2001; Friocourt et al., 2001; Ringstad et al., 2001; Vinatier et al., 2006). To examine VGLUT2-pH recycling in a VGLUT2 background, we used a thalamic culture preparation (Kanagasabapathi et al., 2011; Weston et al., 2011). We first verified that the majority of synapses in hippocampal cultures express VGLUT1 by co-localization of antibody to VGLUT1 or VGLUT2 with antibody to the common SV protein synaptophysin (**Figure 3**). In cultured hippocampal neurons, 92.00 ± 5.05% of synaptophysin^+^ puncta co-localize with antibody to VGLUT1, while only 28.67 ± 5.15%, co-localize with antibody against VGLUT2, ^∗∗^*p* < 0.01 (**Figure 3A**). In thalamic neurons in culture, 80.00 ± 2.28% of synaptophysin^+^ puncta co-localize with antibody against VGLUT2, while VGLUT1 is almost undetectable, 0.80 ± 0.49%, ^∗∗^*p* < 0.01 (**Figure 3B**), consistent with previous studies (Moechars et al., 2006; Weston et al., 2011). As was the case in hippocampal cultures, VGLUT2-pH expressed in thalamic cultures undergoes endocytosis at a slower rate than VGLUT1-pH (**Figure 3C**). The rate of post-stimulus endocytosis of VGLUT2-pH (τ = 54.32 ± 9.34 s) is significantly slower than VGLUT1-pH (τ = 24.89 ± 4.18 s, ^∗^*p* < 0.05) (**Figure 3C**, left and right panels). During stimulation, the decay from peak fluorescence is also smaller for VGLUT2-pH (25.41 ± 3.32%) than VGLUT1-pH (42.02 ± 6.85%, ^∗^*p* < 0.05) (**Figure 3C**, left and middle panels). In addition, the rate of VGLUT2-pH exocytosis [(ΔF/F_0_)/s: 0.0162 ± 0.0008] is slower than that of VGLUT1-pH (0.0205 ± 0.0012, ^∗^*p* < 0.05) (**Figure 3D**, left and middle panels). The size of the RP is similar for VGLUT2-pH (49.93 ± 1.34% of total pool) and VGLUT1-pH (49.10 ± 2.16%) (**Figure 3D**, left and right panels). Total expressed protein levels, measured with 50 mM NH_4_Cl, are not significantly different for the two isoforms in thalamic cultures (data not shown). Thus, VGLUT1-pH and VGLUT2-pH retain their intrinsically distinct recycling kinetics in hippocampal or thalamic neurons. Subsequent experiments were performed in hippocampal cultures to allow use of standard protocols.

### VGLUT C-Termini Direct Isoform-Specific Trafficking

**FIGURE 4:**
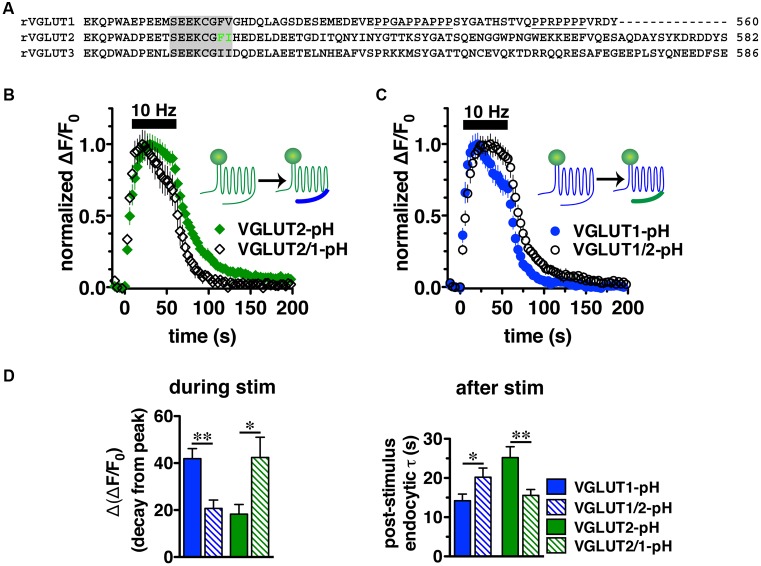
Vesicular glutamate transporter C-termini direct isoform-specific trafficking. **(A)** Alignment of putative targeting sequences in the C-termini of rat VGLUT1, 2, and 3 downstream of the last transmembrane spanning region. Dileucine-like motifs (shaded) are conserved among the three isoforms. The F_518_I_519_ motif of VGLUT2 is in green. Polyproline domains (underlined) are present only in VGLUT1. **(B)** Swapping the C-terminal tails of the two VGLUT-pHs produces chimeric VGLUT2/1-pH and VGLUT1/2-pH (insets). Replacing the VGLUT2 C-terminus with the VGLUT1 C-terminus results in a faster time course of fluorescence change of the chimeric VGLUT2/1-pH (black, *n* = 8) relative to wild-type (WT) VGLUT2-pH (green, *n* = 9). **(C)** Conversely, replacing the VGLUT1 C-terminus with the VGLUT2 C-terminus results in a slower time course of fluorescence change of the chimeric VGLUT1/2-pH (black, *n* = 8) relative to WT VGLUT1-pH (blue, *n* = 11). **(D)** Quantification shows that the extent of fluorescence decay from peak fluorescence [Δ(ΔF/F_0_)] during stimulation (left panel) is greater and the rate of endocytosis after stimulation (right panel) of VGLUT2/1-pH (hatched green) is faster than WT VGLUT2-pH (green), similar to that of WT VGLUT1-pH (blue). Likewise, fluorescence decay for the chimeric VGLUT1/2-pH (hatched blue) is lower than WT VGLUT1-pH (blue), and more similar to WT VGLUT2-pH (green). Data are means ± SEM of ΔF/F_0_, acquired every 3 s, normalized to total fluorescence and to peak fluorescence in each trace during stimulation. Data are from at least 18 synapses per coverslip from 8 to 11 coverslips from at least three independent cultures, ^∗^*p* < 0.05, ^∗∗^*p* < 0.01.

We next sought to characterize the molecular determinants that direct isoform-specific VGLUT trafficking. All three VGLUT isoforms exhibit a high degree of sequence homology in their 12 transmembrane domains, but diverge considerably at the cytoplasmic N- and C-termini (**Figure 4A**) (Takamori, 2006; Voglmaier and Edwards, 2007). The most notable difference is that the C-terminus of VGLUT1 contains two proline rich domains (underlined), not present in VGLUT2 or -3, one of which interacts with the endocytic protein endophilin (De Gois et al., 2006; Vinatier et al., 2006; Voglmaier et al., 2006) (**Figure 4A**). We recently found that VGLUT1 trafficking is directed by regulatory motifs from both N- and C-terminal regions, whereas VGLUT2 trafficking depends mainly on *cis*-regulatory elements in the C-terminus (Foss et al., 2013). Differences in the cytoplasmic C-termini have also been suggested to contribute to the distinct electrophysiological properties of the two VGLUTs (Fremeau et al., 2004a; Weston et al., 2011).

To directly test whether the C-terminal regions of VGLUT1 and 2 are sufficient to drive recycling, we swapped the C-terminal tails of the two isoforms to produce a chimeric VGLUT1-pH reporter containing the VGLUT2 C-terminus (VGLUT1/2-pH), and VGLUT2-pH with the VGLUT1 C-terminus (VGLUT2/1-pH). We transfected the resulting chimeric constructs into rat hippocampal neurons. Upon stimulation at 10 Hz for 60 s, we found that the chimeric VGLUT2/1-pH (black diamonds) recycles faster than WT VGLUT2-pH (green) (**Figure 4B**), and chimeric VGLUT1/2-pH (black circles) recycles more slowly than WT VGLUT1-pH (blue) (**Figure 4C**). The chimeric VGLUT2/1-pH exhibits a faster rate of post-stimulus endocytosis (τ = 15.58 ± 1.48 s, green hatched) than WT VGLUT2-pH (τ = 25.20 ± 2.77 s, green solid, ^∗∗^*p* < 0.01), and is as fast as WT VGLUT1-pH (τ = 14.18 ± 1.73 s, blue solid) (**Figure 4D**, right panel). The fluorescence decay from peak [Δ(ΔF/F_0_)] during stimulation is also significantly greater for chimeric VGLUT2/1-pH (42.41 ± 8.60% from peak, green hatched) than WT VGLUT2-pH (18.26 ± 4.13% from peak, green solid, ^∗^*p* < 0.05), and is similar to WT VGLUT1-pH (41.90 ± 4.31% from peak, blue solid) (**Figures 4C,D**, left panel). Similarly, the chimeric VGLUT1 with the VGLUT2 tail (VGLUT1/2-pH, black circles) recycles more slowly than WT VGLUT1-pH (blue) (**Figure 4C**). VGLUT1/2-pH internalizes more slowly than WT VGLUT1-pH after stimulation (τ = 20.20 ± 2.35 s, blue hatched, ^∗^*p* < 0.05) (**Figure 4D**, right panel). The fluorescence also decays from peak during stimulation to a lesser degree [Δ(ΔF/F_0_) = 20.69 ± 3.58% from peak, blue hatched] (**Figure 4D**, left panel). These results show that regulatory elements in the C-termini are sufficient to drive differential trafficking of the transporter proteins.

### A C-Terminal Dileucine-Like Sorting Motif Is Essential for VGLUT2 Recycling

**FIGURE 5:**
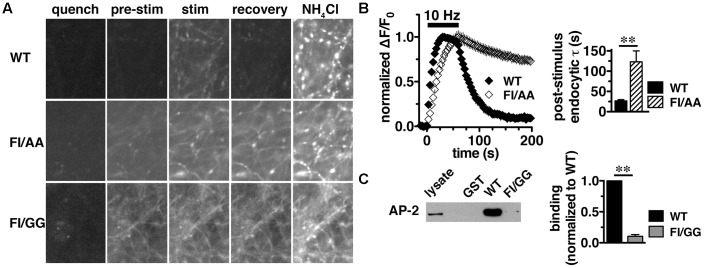
A C-terminal dileucine-like motif is essential for VGLUT2 synaptic targeting and recycling. **(A)** Representative images of neurons expressing WT, FI/AA or FI/GG VGLUT2-pH in Tyrode’s buffered with MES pH 5.5 to quench surface fluorescence (quench); at rest, pH 7.4 (pre-stimulus); during 10 Hz 60 s stimulation, near peak fluorescence (stim); during recovery after termination of the stimulus (recovery); and upon alkalinization in 50 mM NH_4_Cl to visualize total pHluorin levels. The fluorescence of WT VGLUT2-pH increases upon stimulation and quickly recovers to baseline after stimulation stops (top panels). Mutation of the F_518_I_519_ residues to either alanine (FI/AA) or glycine (FI/GG) results in more protein stranded at the cell surface (middle and bottom panels). A fraction of FI/AA in puncta responds to action potential stimulation with an increase in fluorescence, but fails to recover (middle panels). FI/GG mutation severely disrupts synaptic targeting, with fluorescence diffused along neuronal processes, with virtually no response to electrical stimulation (bottom panel). Scale bar, 10 μm. **(B)** *Left panel:* Time course of changes in fluorescence intensity of neurons expressing either WT or FI/AA VGLUT2-pH, normalized to peak. Upon stimulation, WT exhibits a rapid increase in fluorescence consistent with exocytosis, followed by rapid recovery to baseline after the termination of stimulation (black). The small fraction of FI/AA that appears punctate responds to stimulation but fails to recover (open symbols). *Right panel:* The post-stimulus endocytic rate of FI/AA is dramatically slower than that of WT (WT, τ = 26.76 ± 2.963 s, *n* = 6; FI/AA, τ = 123.2 ± 26.81 s, *n* = 7, ^∗∗^*p* < 0.01). Data in **(B)** are means ± SEM of ΔF/F_0_, acquired every 3 s, normalized to total fluorescence and to the peak fluorescence in each trace during stimulation. Data are from at least 41 synapses per coverslip from 6 to 8 coverslips from at least two independent cultures. **(C)** VGLUT2 interacts with the clathrin adaptor protein adaptor protein (AP)-2 through its dileucine-like motif. A GST fusion of the WT VGLUT2 C-terminus specifically pulls down AP-2 from the rat brain lysates, but mutation of F_518_I_519_ to GG disrupts the interaction. *Left panel:* A representative immunoblot of bound proteins detected by antibody to AP-2. *Right panel:* The averaged quantified band intensity from three independent experiments, ^∗∗^*p <* 0.01.

We previously reported that the targeting of VGLUT2 to SVs depends almost entirely on the C-terminus. To further dissect the role of the C-terminal signal in regulating VGLUT2-pH recycling, we mutated the hydrophobic F_518_I_519_ residues in the dileucine-like motif to alanine (FI/AA) or glycine (FI/GG), and monitored recycling of the mutants FI/AA VGLUT2-pH and FI/GG VGLUT2-pH by real time imaging in transfected rat hippocampal neurons (**Figure 5A**). Even at rest (pre-stimulus), the synaptic targeting of FI/AA VGLUT2-pH is disrupted, with significantly more protein trapped at the cell surface (9.61 ± 0.98% of total protein) than WT (∼2.4%). FI/GG mutation results in more cell surface expression (29.22 ± 1.77% of total protein), confirming what we previously reported (Foss et al., 2013). Glycine mutants of VGLUT2 behave like the analogous mutations of dileucine-like motifs in VGLUT1-pH and VGAT-pH, showing a more severe defect in synaptic localization than alanine mutation (Foss et al., 2013; Santos et al., 2013). Time-lapse images show that the fluorescence intensity of WT VGLUT2-pH quickly increases in response to stimulation, and then rapidly recovers to baseline once stimulation stops, as the vesicle internalizes and reacidifies (**Figure 5A**, top panels). However, the two mutants exhibit severely impaired endocytosis and fail to internalize even after 5 min of recovery (**Figure 5A**, middle and lower panels). Alkaline trapping with 50 mM NH_4_Cl reveals similar levels of total protein expression. FI/GG nearly abolishes VGLUT2 recycling from the cell surface; the mutant exhibits a diffuse, non-synaptic distribution, which barely responds to stimulation (**Figure 5A**, lower panels). The small fraction of FI/AA VGLUT2-pH that appears punctate exhibits an increase in fluorescence upon stimulation (**Figure 5A**, middle panels), but fails to internalize (**Figure 5B**, left panel). FI/AA and FI/GG VGLUT2-pH fluorescence after stimulation is quenchable to baseline by application of pH 5.5 Tyrode’s solution in the bath (data not shown). Complete surface quenching indicates the mutant transporters are trapped on the cell surface with the C-terminal pHluorin facing the extracellular space, further indicating that the mutants fail to recycle from the plasma membrane. Accordingly, the rate of post-stimulus endocytosis of FI/AA VGLUT2-pH is dramatically slowed (WT, τ = 26.76 ± 2.963 s; FI/AA, τ = 123.2 ± 26.81 s, ^∗∗^*p* < 0.01) (**Figure 5B**, right panel). These results demonstrate that replacement of the dileucine-like signal, encoded by residues F_518_I_519_, by either AA or GG in the VGLUT2 C-terminus essentially eliminates endocytosis of the transporter protein.

### VGLUT2 Interacts with the Clathrin Adaptor Protein AP-2 through Its Dileucine-Like Motif

Dileucine-like motifs are thought to drive clathrin mediated endocytosis by recruiting APs such as AP-2 (Takei et al., 1996; Robinson and Bonifacino, 2001). Since the dileucine-like residues F_518_I_519_ play an essential role in the synaptic targeting and internalization of VGLUT2 as shown above, we next examined whether there is a specific interaction of the dileucine-like motif with AP-2 by GST pull-down assays. We generated GST fusions of the C-terminus from WT, FI/AA, and FI/GG VGLUT2. Equal amounts of fusion proteins were bound to glutathione beads, incubated with rat brain lysates, and bound proteins were detected by immunoblotting with antibody against AP-2. The WT VGLUT2 fusion specifically pulls down AP-2, whereas mutation of the F_518_I_519_ residues to either AA or GG essentially eliminates the interaction (**Figure 5C** and data not shown). This is consistent with the impaired endocytosis of these mutations observed in live cell imaging (**Figures 5A,B**).

### VGLUT2-pH Undergoes Recycling by a Brefeldin A-Sensitive Pathway

Adaptor protein-2 dependent clathrin mediated endocytosis is a well-established pathway for receptor mediated endocytosis from the plasma membrane (Bonifacino, 2014) and AP-2 plays a crucial role in the fast endocytosis of SV proteins, including VGLUT1 (Takei et al., 1996; Granseth et al., 2006; Voglmaier et al., 2006; Kim and Ryan, 2009; Saheki and De Camilli, 2012; Foss et al., 2013; Hollopeter et al., 2014; Kononenko et al., 2014). However, alternate APs, such as AP-1 and AP-3, also function in SV recycling (Polo-Parada et al., 2001; Nakatsu et al., 2004; Seong et al., 2005; Scheuber et al., 2006; Voglmaier et al., 2006; Danglot and Galli, 2007; Tsytsyura et al., 2007; Kim and Ryan, 2009; Glyvuk et al., 2010; Cheung and Cousin, 2012; Foss et al., 2013). AP-1 and AP-3 have been shown to mediate SV formation from endosomes and cisternae that form during activity-dependent bulk endocytosis (Faundez et al., 1998; Glyvuk et al., 2010; Cheung and Cousin, 2012; Cousin, 2014). Since the slower pathway of SV recycling through bulk endocytosis can be induced by strong (prolonged or high frequency) stimulation (Heuser and Reese, 1973; Richards et al., 2000; de Lange et al., 2003; Cousin, 2009), we first applied a prolonged stimulation paradigm of 1500 action potentials at 5 Hz to hippocampal neurons expressing either VGLUT1-pH or VGLUT2-pH.

**FIGURE 6:**
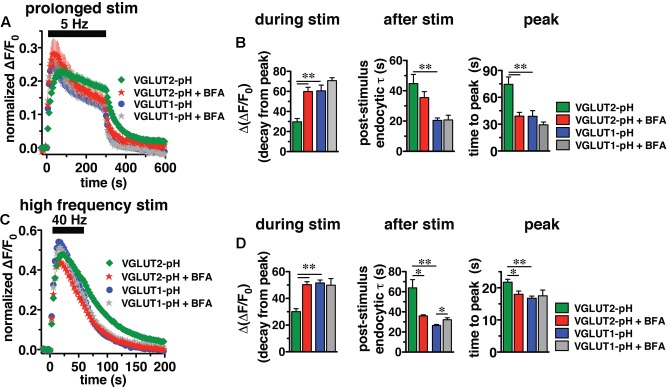
Brefeldin A (BFA) selectively affects VGLUT2 recycling. **(A)** The time course of fluorescence changes of VGLUT1-pH (blue) and VGLUT2-pH (green) expressed in hippocampal neurons, acquired every 6 s, in response to prolonged stimulation at 5 Hz for 5 min are differently affected by BFA. **(B)** *Left panel:* In the presence of BFA (10 μg/ml), VGLUT2-pH fluorescence changes are selectively accelerated. During stimulation, the extent of VGLUT2-pH fluorescence decay from the peak [Δ(ΔF/F_0_)] (green, 29.68 ± 3.25% from peak, *n* = 11) is less than VGLUT1-pH (blue, 60.59 ± 5.63% from peak, *n* = 13, ^∗∗^*p* < 0.01). BFA treatment significantly increases the amount of VGLUT2-pH fluorescence decay from peak during stimulation (red, 59.84 ± 4.25% from peak, *n* = 10), without affecting the amount of VGLUT1-pH decay during stimulation (gray, 70.73 ± 2.89%, *n* = 8). *Middle panel:* VGLUT2-pH also exhibits a slower rate of post-stimulus endocytosis (green, τ = 44.76 ± 6.06 s) than VGLUT1-pH (blue, τ = 20.43 ± 1.63 s, ^∗∗^*p* < 0.01) after prolonged stimulation. The post-stimulus rate of VGLUT2-pH endocytosis is faster in neurons treated with BFA (red, τ = 35.48 ± 3.97 s) than control (green) but is not statistically significant. BFA does not change the rate of VGLUT1-pH endocytosis (gray, τ = 20.75 ± 3.11 s). *Right panel:* While the peak fluorescence level is similar for the two VGLUT-pHs in response to prolonged stimulation, VGLUT2-pH fluorescence peaks more slowly (green, at 74.50 ± 8.27 s) than VGLUT1-pH (blue, at 38.77 ± 6.32 s, ^∗∗^*p* < 0.01). In the presence of BFA, VGLUT2-pH fluorescence reaches peak value at 39.00 ± 4.22 s (red), similar to VGLUT1-pH. BFA treatment does not significantly alter the kinetics of VGLUT1-pH (time to peak 29.25 ± 3.09 s). **(C)** The kinetics of fluorescence change of VGLUT2-pH (green) in response to high frequency stimulation at 40 Hz for 60 s, acquired every 3 s, is different from that of VGLUT1-pH (blue). **(D)** *Left panel:* The VGLUT2-pH fluorescence decay from the peak [Δ(ΔF/F_0_)_,_ green, 30.08 ± 2.16% from peak, *n* = 12] during 40 Hz stimulation is significantly smaller than VGLUT1-pH (blue, 51.53 ± 2.24% from peak, *n* = 12). The decay of VGLUT2-pH during stimulation is significantly increased by BFA treatment (red, 50.30 ± 2.18% from peak, *n* = 8, ^∗∗^*p* < 0.01), whereas that of VGLUT1-pH is not significantly affected by BFA (gray, 49.86 ± 4.96% from peak, *n* = 6). *Middle panel:* VGLUT2-pH exhibits a slower rate of post-stimulus endocytosis (green, τ = 63.81 ± 8.25 s) than VGLUT1-pH (blue, τ = 26.43 ± 1.06 s). BFA significantly accelerates the rate of VGLUT2-pH endocytosis after stimulation (red, τ = 35.99 ± 1.08 s), but slightly decreases that of VGLUT1-pH (gray, τ = 32.13 ± 2.04 s). *Right panel:* VGLUT2-pH fluorescence peaks more slowly (21.75 ± 0.91 s) than VGLUT1-pH (16.75 ± 0.69 s, ^∗∗^*p* < 0.01). BFA treatment shifts the time course of fluorescence changes of VGLUT2-pH (red, time to peak: 17.63 ± 0.98 s) toward that of VGLUT1-pH. The time to peak of VGLUT1-pH fluorescence is not significantly affected by BFA (gray, 17.50 ± 1.80 s) (right panel). Traces in **(A,C)** are means ± SEM of ΔF/F_0_ normalized to total fluorescence. Bar graphs in **(B,D)** are quantifications of fluorescence decay during stimulation and endocytic rate after stimulation. Data are from at least 38 boutons per coverslip from 6 to 12 coverslips from at least three independent cultures, ^∗^*p* < 0.05.

In response to 5 Hz 5 min stimulation, VGLUT2-pH recycles differently than VGLUT1-pH (**Figure 6A**, green vs. blue, right panel). The rate of post-stimulus endocytosis is slower for VGLUT2-pH (green, τ = 44.76 ± 6.06 s) than VGLUT1-pH (blue, τ = 20.43 ± 1.63 s, ^∗∗^*p* < 0.01) (**Figure 6B**, middle panel). The extent of fluorescence decay from peak [Δ(ΔF/F_0_)] during stimulation is also significantly less for VGLUT2-pH (green, 29.68 ± 3.25% from peak) than VGLUT1-pH (blue, 60.59 ± 5.63% from peak, ^∗∗^*p* < 0.01) (**Figure 6B**, left panel). In addition, the peak fluorescence level, when the rate of exocytosis and endocytosis are equivalent, occurs later for VGLUT2-pH (green, 74.50 ± 8.27 s) than VGLUT1-pH (blue, 38.77 ± 6.32 s, ^∗∗^*p* < 0.01) (**Figure 6B**, right panel), while the average fluorescence level at the peak is not significantly different for VGLUT2-pH (green, ΔF/F_0_ = 0.24 ± 0.018) and for VGLUT1-pH (blue, 0.26 ± 0.027) (**Figure 6A**). Thus, VGLUT2 recycles more slowly than VGLUT1-pH in response to 1500 action potentials at 5 Hz.

We next examined the recycling of VGLUT2-pH and VGLUT1-pH in the presence of BFA, an inhibitor of the ADP ribosylation factor 1 GTP exchange factor (ARF-GEF) that, in turn, blocks the activity of AP-1 and AP-3 (Donaldson et al., 1992; Helms et al., 1993; Drake et al., 2000; Pagano et al., 2004; Newell-Litwa et al., 2007). Surprisingly, BFA treatment speeds the recycling of VGLUT2-pH (**Figure 6A**, red). In the presence of BFA, VGLUT2-pH exhibits a rapid increase in fluorescence upon stimulation, reaching the peak value in 39.00 ± 4.22 s (red), more similar to VGLUT1-pH (blue) (**Figure 6B**, right panel). After stimulation, the rate of VGLUT2-pH endocytosis appears moderately faster in BFA-treated neurons (red, τ = 35.48 ± 3.97 s) compared to control (green), but the difference does not reach statistical significance (**Figure 6B**, middle panel). During stimulation, VGLUT2-pH fluorescence decay from the peak is greater in the presence of BFA (red, 59.84 ± 4.25% from peak), compared to vehicle control (green, ^∗∗^*p* < 0.01) (**Figure 6B**, left panel). On the other hand, BFA does not significantly change the response of VGLUT1-pH either during or after 5 Hz 5 min stimulation (**Figures 6A,B**, blue vs. gray). These results suggest that VGLUT2 trafficking depends to a greater degree on AP-1 and/or AP-3 than VGLUT1.

Adaptor protein-1 and AP-3 are involved in activity-dependent bulk endocytosis induced by high frequency stimulation (Clayton et al., 2008; Wenzel et al., 2012). We therefore challenged the neurons using a 40 Hz 60 s (2400 action potential) stimulus (**Figure 6C**). After stimulation, the endocytic rate of VGLUT2-pH (green, τ = 63.81 ± 8.25 s) is significantly slower than that of VGLUT1-pH (blue, τ = 26.43 ± 1.06 s, ^∗∗^*p* < 0.01) (**Figure 6D**, middle panel). During stimulation, the extent of fluorescence decay from the peak [Δ(ΔF/F_0_] is significantly less for VGLUT2-pH (green, 30.08 ± 2.16% from peak) than VGLUT1-pH (blue, 51.53 ± 2.24%, ^∗∗^*p* < 0.01) (**Figure 6D**, left panel). Additionally, the fluorescence intensity of VGLUT1-pH reaches the peak within 16.75 ± 0.69 s, then quickly recovers to baseline (blue). The fluorescence intensity of VGLUT2-pH also rapidly increases in response to stimulation, but peaks later (green, 21.75 ± 0.91 s) than VGLUT1-pH (^∗∗^*p* < 0.01) (**Figure 6D**, right panel).

We next examined whether VGLUT2-pH recycling in response to high frequency (40 Hz 60 s) stimulation is BFA sensitive. As in the case of prolonged stimulation, BFA treatment shifts the kinetics of VGLUT2-pH recycling toward those of VGLUT1-pH (**Figure 6C**). BFA greatly increases the rate of post-stimulus endocytosis of VGLUT2-pH (+BFA, red, τ = 35.99 ± 1.08 s vs. control, green, τ = 63.81 ± 8.25 s, ^∗^*p* < 0.05) (**Figure 6D**, middle panel). The post-stimulus rate of endocytosis of VGLUT1-pH is slightly reduced by BFA (gray, τ = 32.13 ± 2.04 s, vs. blue, ^∗^*p* < 0.05) (**Figure 6D**, right panel). During stimulation, the extent of fluorescence decay from the peak is greater for VGLUT2-pH in BFA-treated neurons (red, 50.30 ± 2.18% from peak) than VGLUT2-pH control (green, 30.08 ± 2.16%, ^∗∗^*p* < 0.01) (**Figure 6D**, left panel). BFA does not significantly affect the amount of fluorescence decay from the peak during stimulation (gray, 49.86 ± 4.96% from peak, vs. blue) of VGLUT1-pH in response to 40 Hz 60 s stimulation (**Figure 6D**, left panel). In addition, the fluorescence intensity of VGLUT2-pH in the presence of BFA (red) peaks at 17.63 ± 0.98 s after the onset of stimulation, appreciably faster than control (green, 21.75 ± 0.91 s, ^∗∗^*p* < 0.01). In the presence of BFA, the rapid increase in VGLUT2-pH fluorescence intensity to peak levels resembles VGLUT1-pH (blue). However, the time to reach peak fluorescence intensity of VGLUT1-pH is not significantly altered by application of BFA (gray, 17.50 ± 1.80 s) (**Figure 6D**, right panel). Taken together, these results show that VGLUT2-pH exhibits different recycling kinetics than VGLUT1-pH both during and after stimulation, and VGLUT2-pH recycling is more sensitive to BFA in response to strong stimulation.

### AP-1 and AP-3 Adaptors Interact with VGLUT2 through Its C-Terminus

**FIGURE 7:**
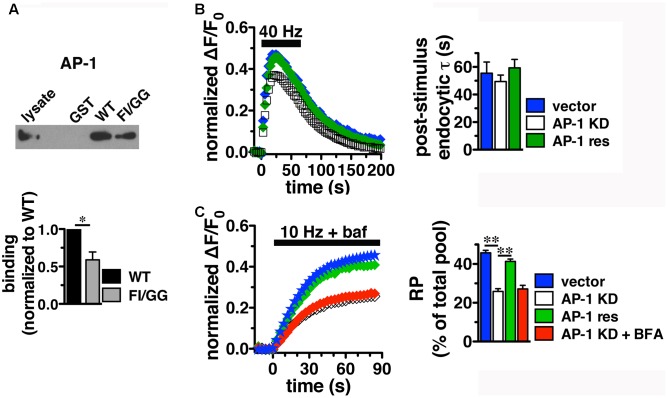
Adaptor protein AP-1 interacts with VGLUT2 and affects the amount of VGLUT2-pH in the RP**. (A)** A GST fusion of the WT VGLUT2-pH C-terminus pulls down AP-1 from rat brain lysate, but the F_518_I_519_ mutation disrupts the interaction. Bound proteins were detected by immunoblotting with AP-1 antibody (anti-adaptin γ). Top panel shows a representative immunoblot, bottom shows the quantification of band intensity from at least three independent experiments, ^∗^*p* < 0.05. **(B)** *Left panel:* Time course of fluorescence changes in boutons from neurons expressing VGLUT2-pH infected with viral particles expressing either vector control (blue) or AP-1γ (open squares), or co-expressing an siRNA resistant 1γ (green). Quantification of peak fluorescence level as a fraction of total pool confirms a reduction with AP-1 KD (white, 37.69 ± 1.60%) compared to vector control (47.63 ± 1.03%, ^∗∗^*p* < 0.01). Co-expression of a construct carrying an siRNA resistant 1γ rescues the peak reduction (green, 46.45 ± 2.48%, *n* = 7). *Right panel:* Neither the post-stimulus endocytic τ_decay_ (right panel, vector, 55.50 ± 8.08 s vs. KD, 49.45 ± 4.69 s) nor fluorescence decay during stimulation [Δ(ΔF/F_0_): vector, 27.45 ± 3.46% vs. AP-1 KD, 32.81 ± 1.65%] is significantly affected by AP-1 depletion. Data in **(B,C)** are means ± SEM of ΔF/F_0_, acquired every 3 s, normalized to total fluorescence. Data are from at least 33 boutons per coverslip from 5 to 7 coverslips from at least two independent cultures. **(C)** Depletion of AP-1 decreases the amount of VGLUT2-pH in the RP, released by 900 action potentials at 10 Hz in the presence of bafilomycin (vector, 45.75 ± 1.34% of total pool, *n* = 22; AP-1 KD, 25.86 ± 1.40%, *n* = 18, ^∗∗^*p* < 0.01). Co-expression of an siRNA resistant 1γ rescues this reduction (41.33 ± 1.24%, *n* = 11, ^∗∗^*p* < 0.01). BFA treatment has no additional effect on AP-1 KD (red, 27.14 ± 1.85%, *n* = 8). The rate of exocytosis in response to 10 Hz 90 s stimulation is not significantly altered by either AP-1 knockdown, rescue, or inhibition by BFA [ΔF/F_0_ (au/s): vector 0.0275 ± 0.0007; AP-1 KD 0.0269 ± 0.0013; AP-1 KD + res 0.0267 ± 0.0021; AP-1 KD + BFA 0.0254 ± 0.0023]. Data in **(C)** are from at least 28 boutons per coverslip from 8 to 22 coverslips from at 4–6 independent cultures.

**FIGURE 8:**
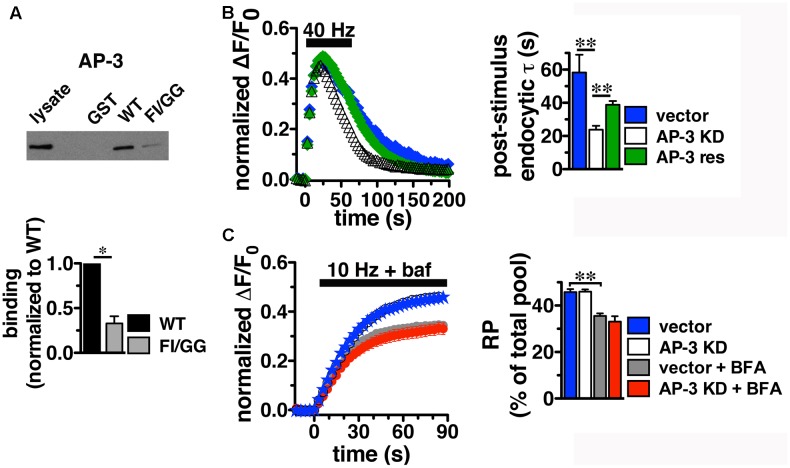
Adaptor protein AP-3 interacts with VGLUT2 and regulates the rate of VGLUT2-pH endocytosis. **(A)** A GST fusion of the WT VGLUT2-pH C-terminus pulls down AP-3 from rat brain lysate. Mutation of the F_518_I_519_ residues of the dileucine-like motif disrupts the interaction. Bound proteins were detected by immunoblotting with antibody against AP-3 (anti-β-NAP). The top panel shows a representative immunoblot, the bottom shows the quantification of WT and mutant band intensities from at least three independent experiments, ^∗^*p* < 0.05. **(B)** AP-3 KD accelerates the recycling kinetics of VGLUT2-pH. *Left panel:* The time course of fluorescence changes in boutons response to 40 Hz stimulation in neurons transfected with VGLUT2-pH and infected with viral particles expressing either vector control (blue) or AP-3δ1 shRNA (open symbols), or co-expressing siRNA resistant δ1 (green). Depletion of AP-3 shifts the time course to the left, the fluorescence peaks earlier with AP-3 KD relative to vector control (vector, 23.50 ± 1.43 s, *n* = 5 vs. AP-3 KD, 20.14 ± 0.55 s, *n* = 7, ^∗^*p* < 0.05). AP-3 KD does not alter the peak fluorescence level during stimulation, as a fraction of the total pool, but it increases the extent of fluorescence decay during stimulation (vector, 27.45 ± 3.46% from peak vs. KD, 54.14 ± 1.71%, ^∗∗^*p* < 0.01). *Right panel:* AP-3 KD speeds the endocytic rate after stimulation (vector, τ = 55.50 ± 8.08 s vs. KD, τ = 20.40 ± 1.71 s, ^∗∗^*p* < 0.01). Expression of an siRNA resistant δ1 rescues the knockdown phenotype (green, τ = 42.14 ± 1.71 s, *n* = 8). Data in **(B,C)** are means ± SEM of ΔF/F_0_, acquired every 3 s, normalized to total fluorescence. Data in **(B)** are from at least 52 boutons per coverslip from 5 to 8 coverslips from at least two independent cultures. **(C)** Depletion of AP-3 does not affect the proportion of VGLUT2-pH in the RP (vector, blue: 45.75 ± 1.34%; AP-3 KD, white: 45.95 ± 1.02%, *n* = 19). Treatment with BFA reduces the proportion of VGLUT2-pH in the RP irrespective of whether neurons were infected with vector control or AP-3δ1 shRNA (vector + BFA, gray: 35.56 ± 1.10%, *n* = 15) and knockdown conditions (AP-3 KD + BFA, red: 33.07 ± 2.35%, *n* = 10), ^∗∗^*p* < 0.01. The rate of exocytosis in response to 10 Hz 90 s stimulation is not significantly altered by AP-3 knockdown, or inhibition by BFA [ΔF/F_0_ (au/s): vector 0.0275 ± 0.0007; AP-3 KD 0.0257 ± 0.0005; vector + BFA 0.0296 ± 0.0009; AP-3 KD + BFA 0.0276 ± 0.0010]. Data in **(C)** are from at least 24 boutons per coverslip from 10 to 22 coverslips from at 4–6 independent cultures.

Since BFA affects both AP-1- and AP-3-mediated trafficking events, we next addressed whether AP-1, AP-3, or both adaptors are involved in VGLUT2 trafficking. First, we asked whether VGLUT2 interacts with AP-1 and AP-3. Although the role of AP-2 in SV protein recycling has been well-characterized, AP-1 and AP-3 have also been shown to play a role (Polo-Parada et al., 2001; Scheuber et al., 2006; Voglmaier et al., 2006; Kim and Ryan, 2009; Glyvuk et al., 2010; Hua et al., 2011; Cheung and Cousin, 2012; Foss et al., 2013). We have demonstrated above that the dileucine-like motif in the C-terminus binds AP-2, and is essential for targeting VGLUT2 to synaptic sites and internalization from the plasma membrane (**Figure 5**). In addition, several SV cargo proteins, including the zinc transporter ZnT3 and the tetanus-insensitive vesicle associated membrane protein TI-VAMP/VAMP7 (Salazar et al., 2004; Hua et al., 2011), interact with AP-3 at dileucine-like motifs. To investigate the biochemical interaction of the VGLUT2 dileucine-like motif with the two alternative endocytic adaptor complexes, we performed GST pull-down assays. Like the AP-2 experiments (**Figure 5C**), GST-fusion proteins of WT VGLUT2 CT and FI/GG mutant CT were bound to glutathione beads, incubated with rat brain homogenate, and then analyzed by immunoblotting with antibodies against AP-1 and AP-3 complexes. WT VGLUT2 CT specifically interacts with AP-1 and AP-3 (**Figures 7A**, **8A**). Mutation of the two hydrophobic resides F_518_I_519_ also decreases binding of VGLUT2 to AP-1 and -3 (**Figures 7A**, **8A**), demonstrating that these residues are important for the interactions.

### VGLUT2 Trafficking Is Differentially Regulated by Adaptor Proteins AP-1 and AP-3

To determine whether the *in vitro* binding of AP-1 and AP-3 has corresponding functional relevance to VGLUT2 recycling at the nerve terminal, we performed shRNA-mediated KD of AP-1 and AP-3. Hippocampal neurons transfected with VGLUT2-pH at the time of plating were infected at DIV 7 with lentivirus containing an shRNA oligonucleotide targeted to AP-1γ or AP-3δ1, along with blue fluorescent protein as a reporter to measure infection efficiency, as described (Foss et al., 2013; Santos et al., 2013). Lentivirus expressing vector alone was used as control. Both the AP-1γ and AP-3δ1 shRNA hairpins used here have previously been shown to specifically deplete the targeted APs with no off-target effects (Asensio et al., 2010; Cheung and Cousin, 2012; Foss et al., 2013; Santos et al., 2013). The AP-1γ shRNA oligonucleotide specifically decreases AP-1 protein complexes from hippocampal neurons at DIV 14–18 to 22.7 ± 8.53% of control. AP-3δ1 shRNA decreases AP-3 complexes to 6.83 ± 1.50% of control, without reduction of the non-targeted APs, as measured by Western analysis (data not shown) (Foss et al., 2013; Santos et al., 2013).

Since we observed the strongest effects of BFA on VGLUT2-pH recycling using high frequency stimulation (**Figure 6C**), we first examine whether AP-1 or AP-3 KD affects VGLUT2-pH recycling in response to a 40 Hz 60 s stimulus. Knockdown of AP-1 decreases the peak level of VGLUT2-pH fluorescence in response to intense stimulation, compared to vector control (**Figure 7B**). Quantification of the peak level as a fraction of the total pool confirms this reduction (vector, 47.63 ± 1.03% vs. AP-1 KD, 37.69 ± 1.60% from peak, ^∗∗^*p* < 0.01). The peak reverts to control levels when an siRNA-resistant AP-1γ is co-expressed to rescue AP-1 function (AP-1 res, 46.45 ± 2.48%) (gray, **Figure 7B**), confirming that the phenotype is specifically due to AP-1 depletion. AP-1 KD does not affect the rate of endocytosis after stimulation (τ_decay_: vector, 55.50 ± 8.08 s vs. KD, 49.45 ± 4.69 s) (**Figure 7B**, right panel). The extent of fluorescence decay from peak during stimulation is also not significantly affected by AP-1 depletion.

During stimulation, the fluorescence peak reflects the balance of endocytosis and exocytosis. Decreases in peak fluorescence can represent an increase in the rate of endocytosis and/or a decrease in the rate exocytosis. The peak fluorescence level can also reflect the extent of exocytosis – the fraction of available VGLUT-pH released by exocytosis in response to high frequency stimulation. To examine how AP-1 KD affects exocytosis of VGLUT2-pH, we used alkaline trapping with the H^+^-ATPase inhibitor bafilomycin to isolate fluorescence changes solely from exocytosis, as described (Sankaranarayanan and Ryan, 2001). To determine the amount of VGLUT2-pH in the RP, we evoked release of the entire RP using a 10 Hz 90 s stimulus, and trapped VGLUT2-pH in the alkaline fluorescent state with bafilomycin (Voglmaier et al., 2006; Ariel and Ryan, 2010). The fraction of VGLUT2-pH in the RP is significantly smaller in neurons depleted of AP-1 (white, 25.86 ± 1.40%) than in the vector control (blue, 45.75 ± 1.34%, ^∗∗^*p* < 0.01) (**Figure 7C**). The reduction of VGLUT2-pH in the RP by the depletion of AP-1 is rescued by co-expression of an siRNA resistant AP-1γ plasmid (green, 41.33 ± 1.24%, ^∗^*p* < 0.01). BFA is a use-dependent inhibitor of both the AP-1 and AP-3 pathways. Pretreatment and stimulation of neurons depleted of AP-1 in the presence of BFA has no additional effect on the amount of VGLUT2 in the RP (red, 27.14 ± 1.85%), suggesting there is no effect of inhibiting AP-3 on RP size. Alkaline trapping also allows us to estimate the rate of exocytosis by measuring the initial slopes of fluorescence change (the first 15 s of stimulation) when neurons were stimulated in the presence of bafilomycin. The rate of exocytosis in response to 10 Hz 90 s stimulation is not significantly altered by either AP-1 KD, rescue, or inhibition by BFA. Thus AP-1 KD affects the extent of exocytosis, but not the rate.

Knockdown of AP-3, however, has different effects on the behavior of VGLUT2-pH. In contrast to AP-1 KD, the depletion of AP-3 accelerates the recycling of VGLUT2-pH in response to intense stimulation; the time course of changes in fluorescence intensity shifts to the left (**Figure 8B**). The fluorescence intensity of VGLUT2-pH increases rapidly upon stimulation and reaches a similar peak level under both control and AP-3 KD conditions. AP-3 KD significantly speeds the rate of post-stimulus endocytosis (vector, τ = 55.50 ± 8.08 s vs. AP-3 KD, τ = 20.40 ± 1.71 s, ^∗∗^*p* < 0.01) (**Figure 8B**). The extent of VGLUT2-pH fluorescence decay from the peak during stimulation is also greater with depletion of AP-3 (white, 54.14 ± 1.71% from peak) than vector control (black, 27.45 ± 3.46% from peak, ^∗∗^*p* < 0.01) (**Figure 8B**). Furthermore, co-expression of an siRNA-resistant AP-3δ1 (Asensio et al., 2010; Santos et al., 2013), rescues the AP-3 KD phenotype, confirming that the effects are specifically due to the loss of AP-3 (**Figure 8B**, green). Alkaline trapping and 10 Hz 90 s stimulation to release the RP shows that KD of AP-3 does not affect the fraction of VGLUT2-pH in the RP (vector control, blue: 45.75 ± 1.34; AP-3 KD, white: 45.95 ± 1.02%) (**Figure 8C**). Addition of BFA to neurons depleted of AP-3 lowers the amount of VGLUT2-pH in the RP (red: 33.07 ± 2.35%), consistent with BFA exerting its effect on RP size through AP-1. The decrease in VGLUT2-pH in the RP with BFA treatment (**Figure 8C**, white) is less than AP-1 KD (**Figure 7C**, white) which may be due to less efficient inhibition, or its acute actions at the nerve terminal, while AP-1 KD may also affect the biosynthetic pathway or endosomal pools located in the cell bodies (Asensio et al., 2010; Larimore et al., 2011; Santos et al., 2013). BFA treatment similarly decreases the amount of VGLUT2-pH in the RP in the control condition (**Figure 8C**, gray, 35.56 ± 1.10%).

Depletion of either AP-3 or BFA treatment does not significantly alter the rate of VGLUT2-pH exocytosis, suggesting the KD phenotypes are not due to a change in exocytosis rate (**Figure 8C**). Together, these results suggest that the decreased peak response with AP-1 KD (**Figures 7B,C**) is likely due to a reduction in available RP vesicles, and not due to altered rates of exo- or endocytosis during stimulation. In contrast, the acceleration of VGLUT2-pH recycling by AP-3 KD (**Figure 8B**) is likely due to increased endocytosis, not a change in exocytosis rate or RP size. Thus, AP-3 can interact with VGLUT2 to modulate its recycling at presynaptic sites.

Taken together, these results show that AP-1 and AP-3 can differentially regulate the trafficking of VGLUT2. Depletion of AP-3 accelerates the recycling of VGLUT2-pH, causing faster internalization of the transporter, while RP size is not affected. Conversely, AP-1 KD does not alter VGLUT2-pH recycling, either in terms of the time required to reach peak fluorescence value or the rate of endocytosis. However, AP-1 KD decreases the fraction of VGLUT2-pH that undergoes exocytosis from the RP.

## Discussion

The expression of VGLUT2 in largely complementary brain regions to VGLUT1 is associated with different physiological properties of synapses in those regions. Previous data suggested that trafficking differences between the two proteins underlie their distinct physiological properties (Fremeau et al., 2001, 2004b; Voglmaier and Edwards, 2007; Weston et al., 2011). Intrinsic sorting signals in VGLUT1 direct its recycling by interacting with the clathrin adaptor protein AP-2 and the endocytic protein endophilin (Voglmaier et al., 2006; Foss et al., 2013; Santos et al., 2014). However, little is known about the mechanisms by which VGLUT2 recycles. It has been increasingly recognized that individual SV proteins use different trafficking motifs to engage highly specialized biochemical mechanisms to regulate their recycling (Salazar et al., 2004; Foss et al., 2013; Kononenko et al., 2013; Santos et al., 2013). Here we find that VGLUT2-pH recycles differently from VGLUT1-pH under several stimulation conditions and in different neuronal cultures, supporting the notion that protein identity, rather than cell type or vesicle membrane composition, governs sorting of SV proteins (Bonanomi et al., 2006; Voglmaier and Edwards, 2007; Cousin, 2017). When exogenously expressed in VGLUT1 or VGLUT2 deficient neurons, physiological properties recorded from these neurons reflect those of the “rescuer” VGLUT isoform, although the VGLUT1 paired-pulse ratio phenotype was only partially recovered in thalamic neurons (Weston et al., 2011). We previously showed that VGAT-pH displays the same recycling profile in both hippocampal and striatal neurons (Santos et al., 2013); here we demonstrate that even the closely related VGLUT isoforms display different behaviors when expressed in identical cellular environments. Endocytosis and exocytosis of VGLUT2-pH are slower than VGLUT1-pH.

The difference in exocytosis rates is consistent with findings that VGLUT2-expressing synapses depress faster than VGLUT1-expressing synapses (Hioki et al., 2003; Fremeau et al., 2004a; Weston et al., 2011). Several factors may underlie synaptic depression, including changes in calcium influx, release site clearance, and depletion of release-ready vesicles (Dittman et al., 2000; Kavalali, 2007; Fioravante and Regehr, 2011). Studies in non-mammalian species show that depressing (phasic) synapses have a smaller RRP than facilitating (tonic) synapses (Millar et al., 2002; Pan and Zucker, 2009). Indeed, here we find that the amount of VGLUT2-pH protein sorted to the RRP is less than VGLUT1-pH. On the other hand, when estimating RRP by measuring charge transfer in hypertonic sucrose, Weston et al. (2011) did not detect a significant difference in the RRP between VGLUT1 and VGLUT2 synapses, although the VGLUT2 RRP was slightly smaller. Charge transfer measurements were noted to depend on the number of synapses recruited, which was variable in the preparation. It should also be noted that here we measure the amount of protein in the RRP of individual synapses, not the glutamate content or the membrane. We find less VGLUT2-pH in the RRP with three applicable approaches, while the amount of VGLUT2-pH in the RP is the same as VGLUT1-pH. The difference is not due to protein expression, since both isoforms show similar levels of total and cell surface expression.

Increasing evidence supports the idea that endocytosis, once thought to be merely a “housekeeping” process to compensate for exocytosis, can be rate-limiting for neurotransmitter release. Endocytosis can determine the recovery from short-term depression by clearing release sites or supplying SVs for reuse (Sudhof, 2004; Wilkinson and Lin, 2004; Ryan, 2006; Kavalali, 2007; Neher, 2010; Hua et al., 2013; Rajappa et al., 2016). Slower endocytosis and decreased protein targeting of VGLUT2 to the RRP could also lead to a decrease in the rate of vesicle filling resulting in decreased glutamate release (Fremeau et al., 2004b; Edwards, 2007). The slower rate of exocytosis and/or endocytosis of VGLUT2 could thus lead to faster synaptic depression in VGLUT2-expressing synapses. Here VGLUT1 and VGLUT2 chimeras demonstrate that *cis*-regulatory elements in the C-termini suffice to drive differential endocytosis of the two isoforms. VGLUT2 lacks the PP domain of VGLUT1 that interacts with endophilin to speed endocytosis, or the contribution to synaptic localization promoted by the additional dileucine-like internalization motifs found in the N-terminus of VGLUT1 (Voglmaier et al., 2006; Foss et al., 2013). Thus, VGLUT1 trafficking is directed by multiple regulatory motifs in both N- and C-termini. In contrast, despite a high level of sequence conservation, VGLUT2-pH recycling shows a remarkably higher degree of dependence on the C-terminal dileucine-like motif. Mutation of the F_518_I_519_ residues in VGLUT2-pH essentially eliminates synaptic targeting and severely disrupts internalization of the protein. Endocytosis of mutant VGLUT2-pH in the few remaining puncta is drastically slowed. Intriguingly, the high dependence of VGLUT2 on the C-terminal dileucine-like motif for endocytosis is more like vesicular transporters for monoamines, GABA, and acetylcholine (VMAT2, VGAT, and VAChT) than the closely related VGLUT1 (Tan et al., 1998; Santos et al., 2001, 2013; Li et al., 2005).

Intracellular targeting and internalization of many SV proteins rely on specific interactions of intrinsic sorting motifs with specialized endocytic adaptors, including clathrin APs, endophilin, and stonin (Bonifacino and Traub, 2003; Ferreira et al., 2005; Li et al., 2005; Diril et al., 2006; Voglmaier et al., 2006; Kelly et al., 2008). We find that VGLUT2 strongly interacts with AP-2 through the key F_518_I_519_ residues. These residues are crucial for both synaptic localization of VGLUT2-pH at steady-state and endocytosis after stimulation. The role of AP-2 in SV recycling is well known, however, substantial SV endocytosis occurs without AP-2 (Gu et al., 2008; Kim and Ryan, 2009; Kononenko et al., 2014; Kononenko and Haucke, 2015). Increasing evidence shows that the alternate adaptors AP-1 and AP-3 are also engaged in SV recycling. These adaptor complexes are enriched at nerve terminals, are present on SVs, and contribute to SV recycling through endosomal intermediates (Faundez et al., 1998; Pagano et al., 2004; Takamori et al., 2006; Glyvuk et al., 2010; Newell-Litwa et al., 2010; Cheung and Cousin, 2012).

We discovered that VGLUT2-pH recycling is sensitive to the AP-1/3 pathway inhibitor BFA. Remarkably, BFA accelerates VGLUT2-pH recycling, shifting its kinetics toward those of VGLUT1-pH. In the presence of BFA, VGLUT2-pH fluorescence decays faster after stimulation, reflecting acceleration of endocytosis. In contrast, recycling of VGLUT1-pH is less affected by BFA. We previously demonstrated that BFA speeds recycling during stimulation of a VGLUT1 mutant that does not bind endophilin. Indeed, this VGLUT1-pH mutant mimics the recycling behavior of VGLUT2. Endophilin interaction is thought to direct VGLUT1 to a faster AP-2 pathway, and away from the slower AP-3 route (Voglmaier et al., 2006). Reformation of SVs directly from the plasma membrane by AP-2 presumably occurs faster than SV re-generation from cisternal intermediates via AP-1 or AP-3 (Royle and Lagnado, 2003; Matthews, 2004; Cheung and Cousin, 2012). Since VGLUT2 does not contain PP domains, we reasoned that VGLUT2 recycling would utilize AP-1/3 to a greater extent than VGLUT1. The differential effects of BFA thus support our hypothesis that VGLUT2 recycles by different mechanisms than VGLUT1.

Biochemical analysis also demonstrates that VGLUT2 binds AP-1 and AP-3. Mutation of the F_518_I_519_ residues substantially disrupts the interactions. AP-1 and AP-3 were shown to be essential for generation of SVs by activity-dependent bulk endocytosis; KD of either AP-1 or AP-3 similarly impaired budding of bulk endosomes visualized by electron microscopy (Cheung and Cousin, 2012). Strikingly, using shRNA-mediated KD and live cell imaging, we uncover different roles for AP-1 and AP-3, confirming that they are not functionally redundant and play specific roles in VGLUT2-pH recycling. AP-3 KD selectively increases the rate of VGLUT2-pH endocytosis, but has no effect on the peak fluorescence level reached during stimulation nor the amount of VGLUT2-pH in the RP. This is consistent with our model of the two parallel pathways of SV reformation (Voglmaier et al., 2006; Voglmaier and Edwards, 2007). AP-3 KD diverts VGLUT2-pH vesicles to a faster pathway, presumably mediated by AP-2.

On the other hand, depletion of AP-1 does not alter kinetics of VGLUT2-pH recycling, however, it reduces the peak fluorescence level. Alkaline trapping demonstrates that AP-1 KD decreases the fraction of VGLUT2-pH in the RP. Thus, AP-1 plays a role in sorting VGLUT2 to the RP. Interestingly, we previously found that while AP-1 KD has no significant effect on the peak response of WT VGLUT1-pH, there is a similar reduction in peak fluorescence level of a VGLUT1-pH mutant that disrupts the C-terminal dileucine-like motif. Thus, N-terminal dileucine-like motifs play a role in sorting mutant VGLUT1-pH to the RP in an AP-1 dependent manner (Foss et al., 2013). However, VGLUT2 trafficking depends almost exclusively on C-terminal sorting signals. Knocking down either AP-1 or AP-3 has no significant effect on the rate of exocytosis, however, AP-1 KD does decrease the extent of VGLUT2-pH exocytosis, represented by a reduction in the fraction of VGLUT2-pH in the RP. Indeed, previous studies show that depletion of AP-1 either by genetic ablation or shRNA-mediated silencing of gene expression impairs SV reformation from endosomal compartments (Glyvuk et al., 2010; Cheung and Cousin, 2012). Recent biochemical analyses in mice lacking AP-1 further show that trafficking of SV proteins to the RP is impaired, and that AP-1 and AP-2 recycling pathways are interdependent (Kratzke et al., 2015).

How APs coordinate to recycle VGLUT2 and other membrane proteins continues to be elucidated. Knockdown of either AP-1 or AP-3 impairs SV reformation from endosomal compartments, suggesting the adaptors may function sequentially (Cheung and Cousin, 2012). The different KD phenotypes described here also suggest that AP-1 and AP-3 may not be competing for the site at the same time or in the same cellular environment. Adaptor proteins are coincidence detectors of cargo proteins and specific lipid environments. For example, AP-2 binds both dileucine motifs and phosphatidylinositol bisphosphate (PIP_2_), which is thought to signal that the cargo protein is at the plasma membrane (Gaidarov and Keen, 1999; Jackson et al., 2010; Kelly et al., 2014). Comparative affinities may also be affected by protein interactions or post-translational modifications of either adaptors or cargo. In the case of aquaporin 4, casein kinase 2 phosphorylation regulates its sequential binding to AP-2 to mediate endocytosis, and then to AP-3 to mediate post-endosomal trafficking (Madrid et al., 2001). We have previously shown that mutation of a predicted phosphorylation motif in the VGLUT1 C-terminus influences its binding to AP-2, but not AP-3 (Santos et al., 2014). The involvement of APs in forms of endocytosis that may not involve clathrin, such as kiss-and-run and ultrafast endocytosis, also remains unresolved (Jockusch et al., 2005; Alabi and Tsien, 2013; Watanabe et al., 2013a,b; Wu et al., 2014; Delvendahl et al., 2016). AP-3 does not have a strict requirement for clathrin (Cowles et al., 1997; Faundez et al., 1998). It should be noted that experiments in this study were performed at room temperature. Ultrafast endocytosis occurs only at physiological temperatures, so differential effects are unlikely to be due to this mechanism. In addition, RP size is not affected by temperature, and differences in physiology between VGLUT1 and VGLUT2 synapses are apparent at room temperature (Fernandez-Alfonso and Ryan, 2004; Weston et al., 2011). But how SV reformation from small endosomes produced by ultrafast endocytosis may relate to SV formation from the larger cisternae produced by activity-dependent bulk endocytosis is not yet clear (Cousin, 2017).

The precise mechanisms that underlie the recycling of SV components merit further exploration. While questions remain about how differences in AP interactions could translate into changes in physiological properties such as synaptic plasticity, SV recycling is remarkably plastic and shapes the response of neurons to sustained stimulation (Kavalali, 2007). VGLUT1 alters the recycling of synaptophysin and VAMP2, in a manner dependent on a PP motif which is not present in VGLUT2, so other SV proteins may also be differentially affected by different regulation of VGLUT1 and 2 trafficking (Pan et al., 2015). Taking into account that the two VGLUTs are differentially expressed in cortical and subcortical glutamatergic pathways, their differential mechanisms presents an opportunity to modulate glutamatergic signaling in specific neuronal populations.

## Author Contributions

HL and SV designed the study. HL, MS, YD, and CP carried out experiments. HL, MS, and SV analyzed the data. HL, MS, and CP prepared the figures. HL and SV discussed the results and wrote the manuscript.

## Conflict of Interest Statement

The authors declare that the research was conducted in the absence of any commercial or financial relationships that could be construed as a potential conflict of interest.
